# Vitreous management in Yamane’s technique for crystalline lens dislocation: anterior vitrectomy or PPV?

**DOI:** 10.1186/s12886-023-03204-9

**Published:** 2023-11-17

**Authors:** Yong Tang, Shiqi Yao, Yanhua Chu, Quanhong Han

**Affiliations:** Tianjin Eye Hospital, Tianjin Key Lab of Ophthalmology and Visual Science, Tianjin Eye Institute, Nankai University Affiliated Eye Hospital, Clinical College of Ophthalmology, Tianjin Medical University, Gansu Road 4, Heping District, Tianjin, 300020 China

**Keywords:** Sutureless intrascleral fixation, Crystalline lens dislocation, Posterior vitreous detachment, Intraocular lens

## Abstract

**Objective:**

To study the postoperative visual outcomes and surgical complications of anterior/pars plana vitrectomy and concurrent Yamane’s IOL fixation for crystalline lens dislocation.

**Methods:**

Fifty-three patients (56 eyes) with crystalline lens subluxation/dislocation were enrolled in this retrospective interventional study. Patients received anterior/pars plana vitrectomy and concurrent Yamane’s IOL fixation. Main outcome measures were postoperative BCVA and surgical complications. Proportion of spontaneous PVD and preoperative undetected retinal holes/degeneration (PURH/D) were recorded.

**Results:**

Twenty-four eyes were treated with anterior vitrectomy (Group AnV) and 32 eyes with pars plana vitrectomy (Group PPV). Overall incidence of PURH/D was 10.7% (6/56). Spontaneous PVD occurred in 68.8% (24/32) in Group PPV. During six months follow-up, one case of postoperative RRD and one case of choroidal detachment occurred in Group AnV. There was no significant difference between anterior vitrectomy and PPV in the final BCVA and postoperative complications.

**Conclusion:**

Anterior or pars plana vitrectomy, which are both applicable in YAMANE technique for crystalline lens dislocation, exhibit similar surgical outcomes. Patient’s age, PVD status and PURH helps to determine the route of vitrectomy. Pediatric patients might be potential candidates for transcorneal vitreolensectomy. For adult, PURH managed with total vitrectomy and intraoperative lase retinopexy might be beneficial to decrease the incidence of postoperative RRD.

## Introduction

In-the-bag implantation of intraocular lens (IOL) might not be possible in the absence of adequate capsular support. Crystalline lens/IOL dislocation are common indications for sutured or sutureless intrascleral fixation of IOL. Since first reported by Yamane in 2017 [1], this flanged Intrascleral fixation of IOL has been widely accepted by ophthalmologists with many detail refinements [2–4]. The dislocated IOL or crystalline lens could be exported through the corneal or sclerocorneal incision in anterior vitrectomy or PPV. Rhegmatogenous retinal detachment (RRD) is an uncommon complication after Yamane’s surgery, but requires extra or repeated operations for retinal re-attachment and leads to irreversible damage of visual function. The overall incidence of RRD after sutured/sutureless scleral-fixated IOL(SFIOL) surgery was 1.7–15.2% and 1.5–4.6%, respectively [5]. A high number of retinal breaks were identified during vitrectomy for retained lens fragments in previous research [6]. Previous study suggested that preservation of posterior hyaloid attachment during vitreolensectomy for crystalline lens dislocation was associated with fewer iatrogenic retinal breaks [7]. Our study was done to compare the visual outcomes and surgical complications between anterior vitrectomy and PPV concurrent with Yamane’s IOL fixation for patients with crystalline lens dislocation. Incidence of preoperative undetected retinal holes (PURH) and proportion of spontaneous PVD were also documented.

## Material and method

In this retrospective, IRB-approved, interventional cohort study, we enrolled 56 cases of crystalline lens dislocation (2019.10–2022.10). 24 eyes were treated with anterior vitrectomy (Group AnV) and 32 eyes with pars plana vitrectomy (Group PPV). All surgical cases received concurrent sutureless flanged intrascleral IOL fixation with Yamane technique with at least 6 months follow-up. The lens is defined as dislocated when it lies completely within the vitreous body or the anterior chamber, whereas it is considered subluxated when it is partially displaced but remains within the lens space. 3 cases of complicated cataract surgery and drop of lens fragment were also included in Group PPV. Cases with open globe injury (OGI), preoperative retinal breaks, RRD or previous intraocular surgery were excluded. This study adhered to the tenets of the Declaration of Helsinki and was approved by the Ethics Committee of Tianjin Eye Hospital. Written informed consent was obtained from all subjects. All methods were carried out in accordance with relevant guidelines and regulations.

### Outcome measures

The primary outcome measure was postoperative BCVA at 6 months and the incidence of postoperative RRD and other surgical complications. Incidence of PURH and the proportion of spontaneous PVD in Group PPV were also recorded. PURH was defined as retinal hole or retinal lattice degeneration detected during the surgery. Spontaneous PVD was defined as no vitreous hyaloid attached at the optic disc and macula after staining with triamcinolone acetonide (TA) during the surgery. Pretreatment clinical features and intraoperative findings were compared to find out whether there are certain characteristics that indicate the attachment of posterior hyaloid. Snellen visual acuity was converted to logarithm of the minimum angle of resolution (logMAR) for analysis. Continuous variables were expressed as the mean ± SD. Categorical variables were expressed as frequencies and percentages. The Mann–Whitney U test and Fisher exact test were used to compare parameters between groups. *P* value less than 0.05 was defined as statistically significant. Statistical analyses were performed using SPSS for Windows software (version26.0, IBM Corp.).

### Surgical technique

A sclerocorneal tunnel/corneal incision was constructed superiorly. The crystalline lens with hard nucleus was extracted from corneoscleral incision which was sealed with 10/0 nylon suture. Crystalline lens with soft nucleus was removed with vitreous cutter or phacoemulsification.

#### Group AnV

Anterior displaced crystalline lens was extracted through the corneal or slcerocorneal incision, and vitreous prolapse around the pupil or incarcerated into the surgical incision was removed with anterior vitrectomy. Peripheral retinal examination was done at the end of in Group AnV, predominantly with binocular indirect ophthalmoscopy (BIO). Retinal weak areas were sealed with delayed retinal photocoagulation at the outpatient department. Three children (5 eyes) with crystalline subluxation were managed with transcorneal vitreolensectomy and YAMANE’s IOL fixation.

#### Group PPV

For posterior dislocated crystalline lens, standard three-port complete vitrectomy was performed with vitreous base shaving (Constellation; Alcon Surgical). PVD induction was mandatory for all surgical cases if posterior hyaloid was still attached as proven by triamcinolone acetonide staining. Peripheral search for PURH/D was then conducted under a chandelier endoilluminator for all patients in Group PPV. All the PURH/D were documented and treated with laser retinopexy during the surgery.

#### Yamane’s intrascleral IOL fixation

A modified Yamane technique was then used for intrascleral fixation of IOL, details was described in previous literature [2]. Corneal main port was placed at 12 o’clock with two side ports at 3- and 9-o’lock. An angled sclerotomy was made through the conjunctiva using a 26/27-gauge needle at 2.0 mm from the limbus at 3 o’clock. A 3-piece IOL was inserted into the anterior chamber (AC) using an injector and the leading haptic was conducted into the lumen of the needle. The trailing haptic was left outside the main port. A second sclerotomy then was made with a puncture needle at 9 o’clock. The trailing haptic was inserted into the lumen of the second needle with intraocular forceps through corneal incision at 12 o’clock. Both haptics were externalized onto the conjunctiva, and the ends were cauterized 1.5 mm to make a flange. The flange of the haptics was pushed back and fixed into the scleral tunnels. At the end of PPV, an air or gas tamponade (C3F8) was used.

## Results

The baseline characteristics, final BCVA, intraoperative and postoperative complications of all cases are presented in Tables 1, 2 and 3, with no significant differences between the two group. Incidence of PURH/D was 10.7% (6/56). Spontaneous PVD was confirmed in 66.8% of patients enrolled in Group PPV. PVD induction was mandatory in the rest cases. Pretreatment clinical features and intraoperative findings were compared to find out whether there are certain characteristics that indicate the attachment of posterior hyaloid (Table 4). The differences of presenting age, comorbidity of Marfan Syndrome and presence of PURH/D were statistically significant between patients with or without spontaneous PVD.

**Table 1:** Baseline demographic and clinical characteristics

Characteristic	Group PPV (32 eyes)	Group AnV (24 eyes)	*P* value
Age (y)	61.03 ± 13.53	50.21 ± 25.95	0.26
Male n(%)	25(73.5)	18(79.2)	0.62
Preoperative CDVA(logMAR)	0.98 ± 0.86	0.83 ± 0.77	0.62
Axial length	23.79 ± 1.62	23.76 ± 1.07	0.37
Cause of dislocation			
Marfan Syndrome	3(9.4)	4(16.7)	0.45
Closed Globe injury n(%)	4(12.5)	3(12.5)	1
Complicated cataract surgery	4(12.5)	0	NA
Comorbidity			
Glaucoma n(%)	5(15.6)	2(8.3)	0.69
Traumatic macular hole n(%)	1(2.9)	0	NA
High Myopia	6(18.8)	4(16.7)	1

*NA* not available

**Table 2:** Intraoperative findings and surgical complications

Characteristic n (%)	Group PPV (*n* = 32)	Group AnV (*n* = 24)
Eyes with PURH/D	5(15.6)	1(4.1)
Intraoperative RRD	1(3.1)	0
Spontaneous PVD	24(75.0)	NA
Tamponade		
Saline	0	24(100)
Air	30(93.8)	0
C3F8	2(6.2)	0

*PURH/D* Preoperative undetected retinal holes/degeneration, *NA* not available

**Table 3:** Postoperative CDVA and surgical complications

Characteristic	Group PPV (*n* = 32)	Group AnV (*n* = 24)	*P* value
CDVA(logMAR)	0.24 ± 0.35	0.21 ± 0.19	0.32
VA ≥ 0.5 n(%)	25(78.1)	19(79.2)	0.93
RRD n(%)	0	1(6.3)	0.43
Choroidal detachment n(%)	0	1(4.8)	0.43
Persistent glaucoma n(%)	2(6.3)	0	0.50
Vitreous hemorrhage n(%)	2(6.3)	2(8.3)	1
Optic capture n(%)	2(5.9)	1(4.2)	1

**Table 4:** Clinical features present in cohorts with and without spontaneous PVD

Clinical Feature n(%)		PVD + (22 eyes, 68.8%)	PVD- (10 eyes, 31.2%)	*P* value
**Age(y)**		66.23 ± 7.91	49.60 ± 16.54	0.003
**Gender**	**M**	18(81.8)	8(80)	1
	**F**	4(18.2)	2(20)	
**Axial length(mm)**		23.74 ± 1.35	24.98 ± 2.39	0.025
**Preoperative BCVA**		0.82 ± 0.73	1.05 ± 0.92	0.635
**PURH**		0	5(50%)	0.001

**Fig. 1:**
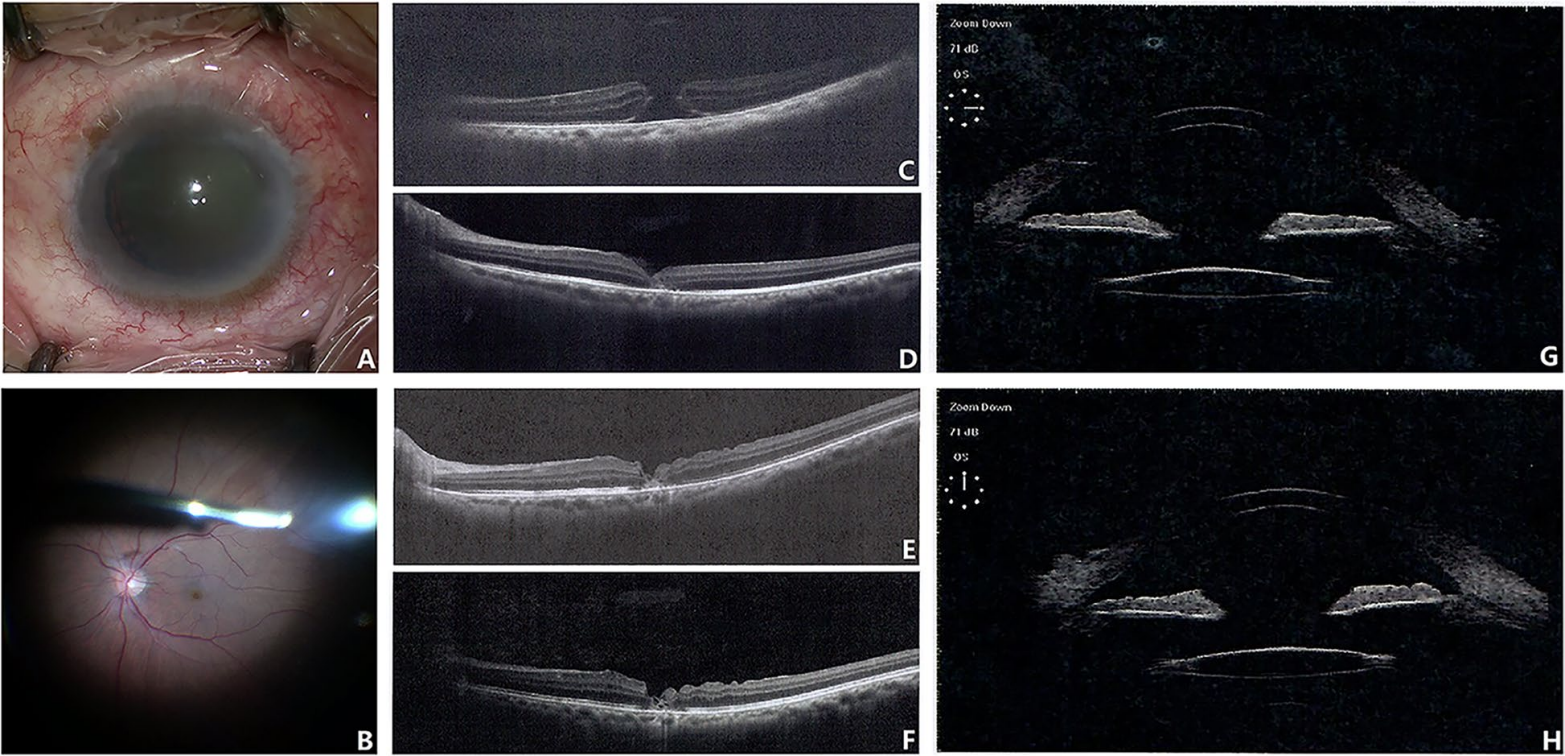
A 48-year-old Chinese man with traumatic crystalline lens subluxation (**A**) and macular hole (**B**) was treated with PPV, internal limiting membrane flap for macular hole closure and YAMANE technique for IOL fixation concurrently. Preoperative OCT showed full thickness macular hole (**C**) in the left eye. One week (**D**), three months (**E**) and six months (**F**) follow-up of OCT showed closure of the macular hole and partial recovery of outer retinal layers. UBM of horizontal and vertical scan (**G**, **H**) at six months showed well-centered IOL with no tilt or decentration. Final BCVA of this patient was 0.7LogMAR

One case of traumatic crystalline lens subluxation and macular hole was treated with PPV, internal limiting membrane flap for macular hole closure and YAMANE technique for IOL fixation concurrently (Fig. 1).

**Fig. 2:**
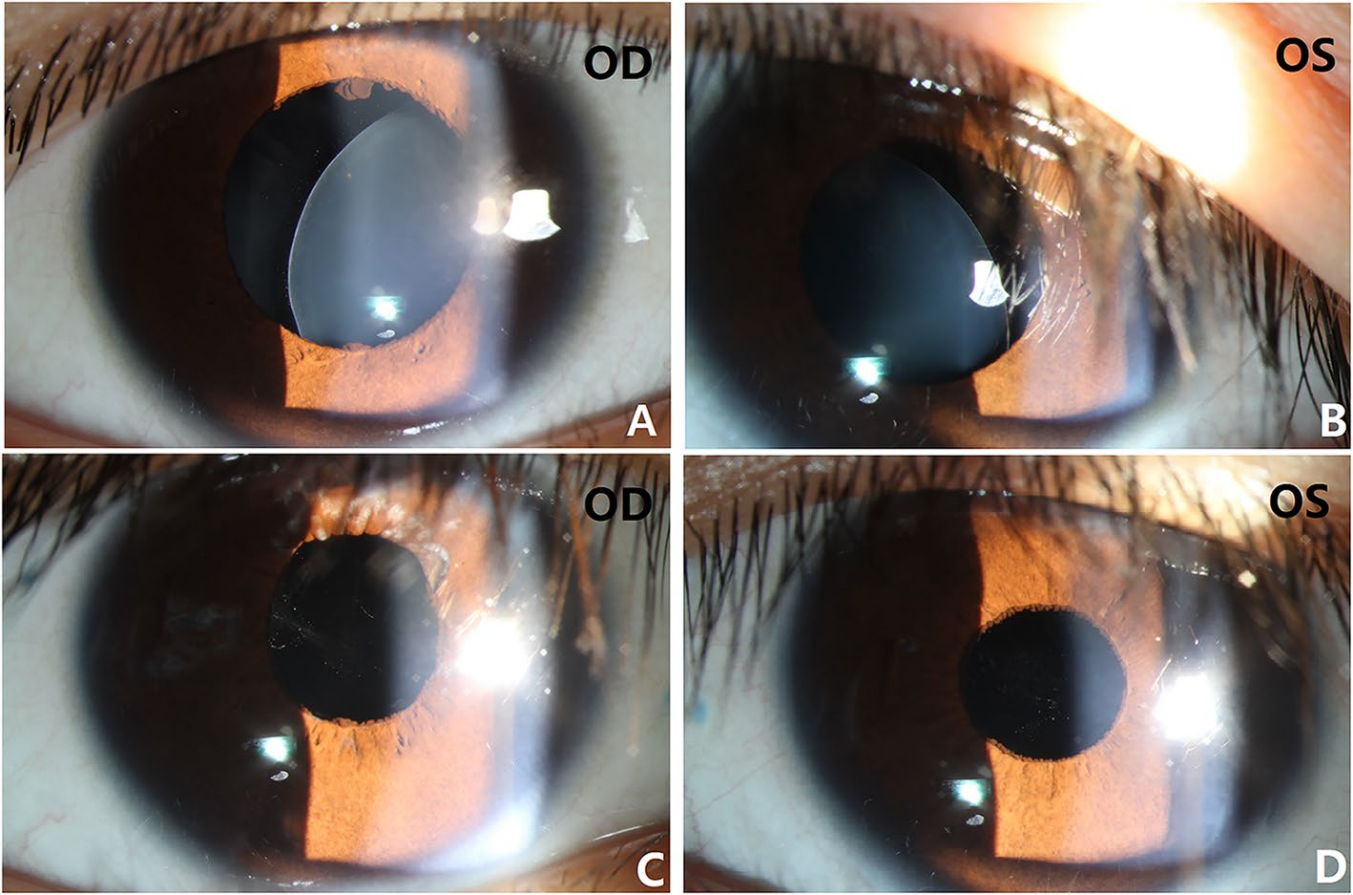
A seven-year-old Chinese boy with lentis ectopia and Marfan Syndrome in both eyes were managed with anterior vitrelensectomy and Yamane’s IOL fixation. Preoperative anterior segment photograph showed inferonasal subluxation of crystalline lens (**A**, **B**) with BCVA 0.5LogMAR in both eyes. One year follow-up showed well-centered IOL (**C**, **D**) and BCVA 0.2LogMAR in both eyes

Three children (5 eyes) with crystalline lens subluxation were managed with transcorneal vitreolensctomy and Yamane’s IOL fixation with improvement of postoperative BCVA and no postoperative complications (Table 5, Fig. 2).

**Table 5:** Characteristics of pediatric patients with lens dislocation

Case No	Age(y)	Gender	Eye	AL(mm)	Comorbidity	Vitrectomy	Preoperative BCVA (LogMAR)	Postoperative BCVA (LogMAR)
1	7	M	OD	22.95	Marfan Syndrome	AnV	0.5	0.2
			OS	22.86		AnV	0.5	0.2
**2**	8	M	OD	24.76	Kawasaki disease	AnV	0.5	0.2
			OS	24.51		AnV	0.7	0.2
3	7	M	OD	25.12	Marfan Syndrome	AnV	0.4	0.18

**Fig. 3:**
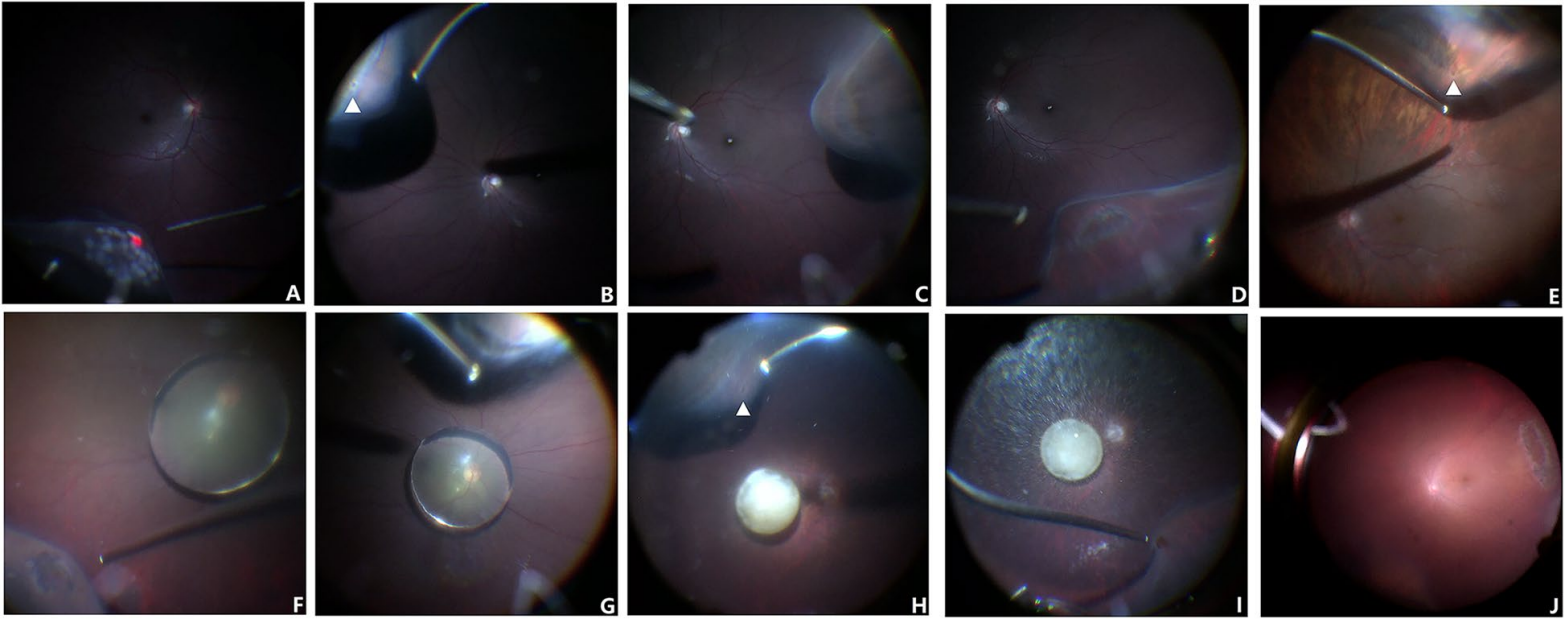
Patients with ectopia lentis and retinal breaks/degeneration. A 27-year-old man with ectopia lentis and Marfan Syndrome in both eyes were managed with PPV and YAMANE’s IOL fixation. Intraoperative fundus examination revealed retinal degeneration in both eyes (**A**, **C**, **D**) and retinal hole (**B**) at the orra serrata in the left eye. Fundus examination from a 58-year-old man with complicated cataract surgery revealed a round retinal hole in the lattice degeneration (**E**). Fundus examination from another 58-year-old man with crystalline lens dislocation showed inferior retinal hole (**F**) with surrounding RPE proliferation and superior lattice degeneration (**G**). A 45-year-old man with crystalline lens dislocation, mature cataract and high myopia was managed with PPV and YAMANE’s IOL fixation. One retinal hole in the suprnasal quadrant (**H**, arrow head) and one retinal tear in the inferotemporal quadrant (**I**) were discovered during the surgery. A 71-year-old man with traumatic crystalline lens subluxation and mature cataract received ECCE, PPV and YAMANE’s IOL fixation. One peripheral retinal tear with RPE proliferation (**J**) was discovered during intraoperative fundus examination under chandelier illumination

Characteristics of the 6 cases with PURH/D (Fig. 3) are listed in Table 6. The mean age in these 6 cases was 51.8 (range 27–71). More retinal breaks/degeneration were detected in Group PPV than in Group AnV(5/34 VS 1/22). In Group PPV, 1 case with retinal break and lattice degeneration (Fig. 3F,G) developed into intraoperative iatrogenic retinal detachment around the lattice degeneration, which was managed with laser retinopexy and air tamponade with retinal reattachment postoperatively. In Group AnV, 1 case of retinal tear at the temporal equator with surrounding RPE proliferation (Fig. 3J) was found intraoperatively in a patient with mature cataract and traumatic crystalline lens subluxation. All patients with retinal breaks/degeneration were treated with prompt or delayed laser retinopexy and no further sequelae.

**Table 6:** Characteristics of patients with intraoperative retinal break(s) and degeneration

Case No	Age(y)	Gender	AL(mm)	Cause of dislocation	Vitrectomy	Spontaneous PVD	Location of Retinal degeneration	No. of Retinal breaks	Type of Retinal breaks	Location of Retinal breaks	Preoperative BCVA (LogMAR)	Postoperative BCVA (LogMAR)
**1**	27	M	25.94	Marfan	PPV	-	Inferotemporal	0	/	/	0.1	0
**2**			26	Marfan	PPV	-	Temporal + Inferotemporal	1	Round hole at orra serrata	Suprnasal	0.1	0.1
**3**	58	M	27.55	Complicated Cataract surgery	PPV	-	Superior	1	Round hole in lattice degeneration	Superior	0	0.1
**4**	58	M	23.98	-	PPV	-	Superior	1	Round hole with RPE proliferation	Inferotemporal	0.1	0.1
**5**	45	M	29.49	Mature Cataract High Myopia	PPV	-	-	2	Round hole	Supranasal	0.7	0.4
									Horseshoe tear	Inferotemporal		
**6**	71	M	23.35	Mature cataract Trauma	AnV	/	-	1	Retinal hole with RPE proliferation	Temporal	-3	0

**Fig. 4:**
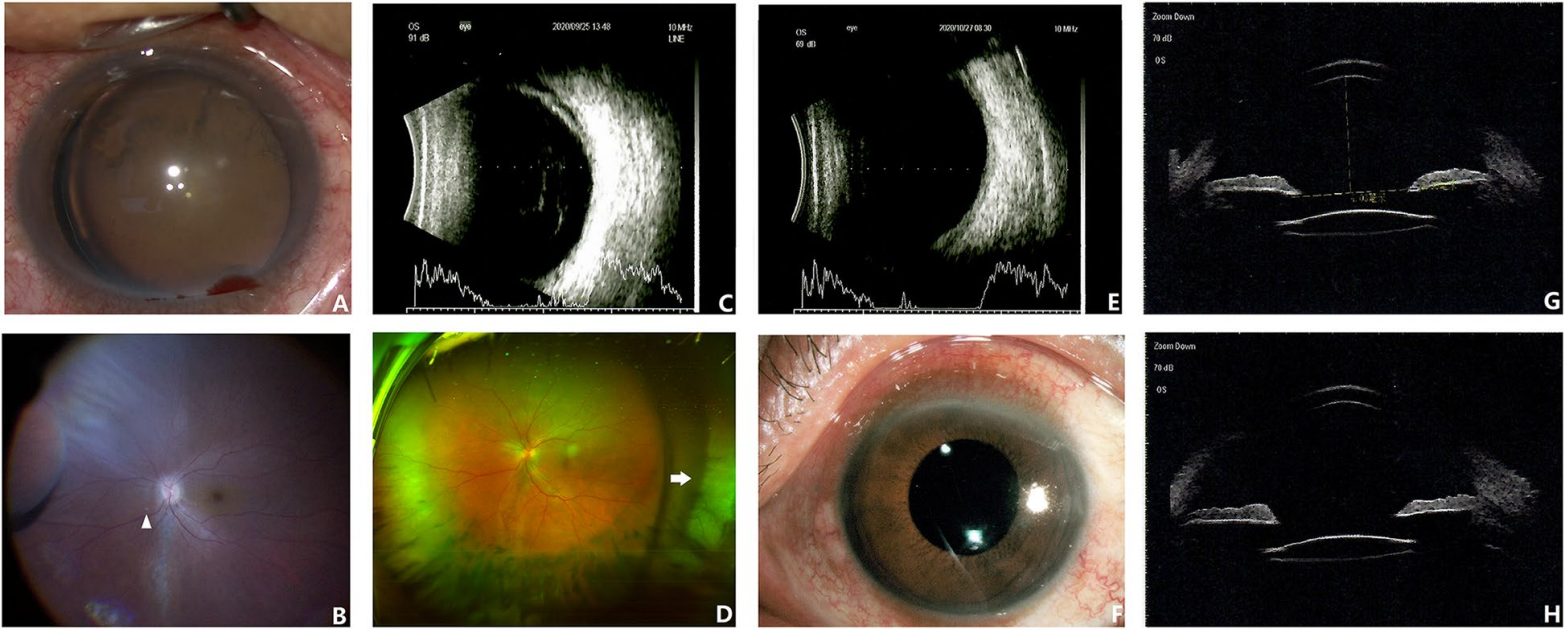
A 48-year-old Chinese man with crystalline lens subluxation (**A**) and secondary glaucoma received anterior vitreolensecotomy and primary YAMANE’s IOL fixation. Intraoperative peripheral retinal search showed no retinal break/degeneration and a tiny lens fragment (**B**, arrow head) dropped in the posterior pole. One week postoperatively the patient returned with blurred vision and blunt pain in the left eye. B-Ultrasound (**C**) and ultrawide fundus photograph (**D**, white arrow) revealed flat choroidal detachment at the inferotemporal periphery, which gradually resolved after topical and periocular administration of corticosteroid two weeks later (**E**). One month follow-up showed clear cornea and well-centered IOL in the left eye(F). UBM of vertical and horizontal scan (**G**, **H**) at six months showed well-centered IOL with no tilt or decentration. Final BCVA of this patient was 0.1LogMAR

One of postoperative RRD was noted in Group AnV and were managed with secondary PPV and silicone oil tamponade. Retinal re-attachment was achieved after silicon oil removal. One case of choroidal detachment was detected in Group AnV (Fig. 4) which resolved in two weeks postoperatively after periocular injection and topical administration of corticosteroid.

## Discussion

Our study demonstrated that postoperative BCVA and incidence of RRD did not differ between anterior vitrectomy or PPV in Yamane’s technique for patients with crystalline lens dislocation. Spontaneous PVD occurred in about two-thirds of patients with crystalline lens dislocation. Total vitrectomy with induction of PVD and laser photocoagulation of intraoperative retinal breaks and lattice degeneration don’t preclude the primary IOL fixation with Yamane’s technique and seems not to increase the risk of postoperative retinal detachment.

Our study showed high proportion (10.7%) of PURH/D in patients with crystalline dislocation. The common causes of crystalline dislocation include Marfan syndrome, ocular trauma and high myopia, which also predispose the patients to retinal breaks/lattice degeneration. One retrospective study of 40 eyes with vitreolensectomy in Marfan Syndrome [8] reported that seven eyes developed retinal detachment at baseline and the postoperative incidence of retinal detachment was 6%. Preoperative fundus evaluation helps to find out these retinal weak areas, while might be hindered by the mature cataract, luxated lens or opacities of other refractive media. Previous research also reported [9] that retinal breaks occurred in approximately one-third of patients with traumatic crystalline lens dislocation and were difficult to observe pre-operation. The pars plana approach has advantages over the trans-limbal approach in that retinal pathology may be treated directly and vitreous removal can be more complete with reduced tractional forces.

Anterior vitrectomy is commonly used by cataract surgeon in circumstance of complicated surgery. Although not as a routine procedure in anterior vitrectomy, peripheral retinal examination does offer us a second chance to find and seal the retinal breaks/degeneration without no further sequela. To lower the incidence of RD after adult cataract surgery in myopic patients, Fan et al. [10] recommended preoperative, intraoperative, and postoperative comprehensive fundus evaluation and laser treatments for suspicious lesions. Therefore, we advocate a similar approach of intraoperative fundus examination, especially in patients with anterior vitrectomy or any other procedures as long as the vitreous was disturbed.

Our series includes the first case report combining YAMANE technique with ILM flap technique to treat patients with crystalline lens subluxation and macular hole. According to our early surgical experience, full thickness closure of macular hole and stable intrascleral fixation of IOL could be achieved in one surgery. Combined with the previous discussion, retinal break/degeneration in crystalline lens dislocation, even macular hole, which could be managed with PPV, doesn’t preclude the primary implantation of IOL with YAMANE’s technique.

As proven with TA staining, spontaneous PVD happened in about two-thirds cases of crystalline lens dislocation in Group PPV, and PVD induction was performed for the rest cases. Comparison between patient with or without PVD showed that younger age, Marfan Syndrome, retinal break/lattice degeneration are indicators of attached posterior hyaloid. As is well known, both the degree of vitreous liquefaction and the prevalence of PVD are age-related [11]. The relative younger age in patients with retinal break/degeneration helps to explain the difference. Ripandelli et al. [12] showed that after cataract surgery, PVD occurred in 77.6% and 87.2% of emmetropic eyes without preoperative lattice degeneration and with lattice degeneration, respectively. Postoperative PVD-induced retinal breaks are associated with increased risk of RRD, which increases multiple folds in eyes having lattice degenerations. To guard against postoperative RRD, attached hyaloid and retinal break/degeneration are indications of PPV for patients with YAMANE technique in our surgical center.

There is still no consensus on preservation or removal of posterior hyaloid in vitreolensectomy. As reported in the study of vitreolensectomy in Marfan Syndrome in 2000 [8], all patients had a complete vitrectomy with removal of the posterior hyaloid face. Other researchers suggested that preservation of posterior hyaloid attachment during vitreolensectomy for crystalline lens dislocation was associated with fewer iatrogenic retinal breaks [8]. In 2020, the largest study in India evaluated the incidence, characteristics, and surgical outcome of RRD after PPV and sutureless SFIOL [5]. The induction of posterior vitreous detachment (PVD) was also not done in any of the cases. The overall incidence of postoperative RRD in their study cohort was 1.7% [5], which is similar to that reported in previous literature with sutured SFIOL [13–15] and in macular surgery [16, 17]. Yet it cannot be neglected that postoperative progression of PVD and peripheral retinal breaks/lattice degeneration leave the patients at high risk of RRD. That’s why we insist that PVD induction is mandatory in PPV——retinal break/degeneration is not a problem, untreated one is.

Our series includes the first case series of sutureless intrascleral fixation of IOL with Yamane technique in pediatric patients with ectopia lentis. Previous research [18] already reported the safety and efficacy of sutureless intrascleral fixated posterior chamber IOL in children with crystalline lens dislocation. Another study comparing the sutured and sutureless fixation of IOL in children suggested that both methods were suitable for the rehabilitation of pediatric aphakia [19]. Anterior trans-limbal approach of vitreolensectomy is preferred in our study for children with crystalline lens dislocation for the following considerations. Infants and children have smaller eyes with different surgical landmarks compared to adult eyes. The posterior trans pars plicata/plana technique can only be considered if the surgeon can safely introduce the instruments without causing an iatrogenic retinal break. PVD is not recommended in children with firmly adherent posterior vitreous like ROP. Forceful creation of a PVD is not only challenging but also carries a high risk of inducing retinal tears [20]. P Sen et al. reported that the incidence of postoperative RRD was 5.7% in children who underwent PPV with sutured scleral-fixed IOL [21]. Sumita et al. [22] reported a 5.5% risk of RD for the first 10 years after cataract surgery in children with no known ocular and systemic anomalies. The risk significantly increases in a male, myopic, and intellectual disabled child. However, PPV might be the reasonable option for children with posterior dislocation of crystalline lens in the vitreous cavity. The need for regular and long-term follow-up after pediatric cataract surgery emphasized in their conclusion is also applicable for children with crystalline lens dislocation.

Although the study is limited by its retrospective nature and small numbers, it brings forth certain surgical aspects: both anterior or pars plana vitrectomy are applicable in YAMANE technique for crystalline lens dislocation with no significant difference in postoperative visual recovery or surgical complications. Incidence of retinal breaks/lattice degeneration was 10.5% in patients with crystalline lens dislocation and spontaneous PVD occurred in about two-thirds of these patients. For pediatric patient with crystalline lens dislocation, anterior vitreolensectomy is preferred with primary YAMANE’s IOL fixation. For adult patients, Preoperative evaluation should focus on the PVD status and peripheral retinal search for potential retinal degeneration/break. PPV is reserved for patients with posterior dislocation of crystalline lens, attached posterior hyaloid (PVD-) and retinal degeneration/break. Intraoperative retinal examination under chandelier illumination or BIO should not be omitted in both anterior vitrectomy and PPV. Routine periodical follow-up might be beneficial for early detection and management for postoperative retinal detachment.

## Data Availability

All the data supporting our findings are contained within the manuscript.
